# Understanding the Association between Musical Sophistication and Well-Being in Music Students

**DOI:** 10.3390/ijerph19073867

**Published:** 2022-03-24

**Authors:** Michel A. Cara, Constanza Lobos, Mario Varas, Oscar Torres

**Affiliations:** 1 Department of Pedagogy, Music Institute, Faculty of Philosophy and Education, Pontifical Catholic University of Valparaíso, Valparaíso 2370688, Chile; mario.varas.v@mail.pucv.cl (M.V.); oscar.torres.r@mail.pucv.cl (O.T.); 2 Department of Sociology, Faculty of Social Sciences, Alberto Hurtado University, Santiago 8340575, Chile; coni.lobosguerrero@gmail.com

**Keywords:** musical sophistication, PERMA-profiler, undergraduate student music teachers, well-being, students’ transition

## Abstract

Quality of life and mental health are topics under discussion in the university environment that pose new educational challenges. Public policy in Chile establishes the need to track students who are starting university and who could find themselves at possible academic risk (Law 20. 903). These transition processes experienced by students therefore need to be guided to improve the students’ quality of life. Using a mixed design, the present study analyzes the association between musical sophistication (Ollen, 2006), students’ well-being, and the performance of first-year students training to be music teachers (*n* = 25). The Ollen Musical Sophistication questionnaire and the Spanish version of the PERMA-profiler, a questionnaire for assessing well-being, were applied. In order to obtain detailed information about learning processes and educational needs, seven interviews were conducted. Results indicate a negative correlation between musical sophistication on the one hand and negative emotions (anxiety and anger) and loneliness on the other. This is reflected in less consistent academic performance, difficulties in identity development, and reduced motivation to face new challenges besides musical learning. We concluded that knowledge and observation of students’ previous musical experience is crucial for understanding and supporting the educational transition process and well-being of student music teachers.

## 1. Introduction

Understanding psychological features of music learning in adults is an important and topical subject, since educational institutions are starting to assess and support students’ well-being, a process accompanied in certain contexts by a legislative framework. Public policy in Chile encourages universities to implement support and levelling mechanisms for students who are just entering teaching courses. Law 20.903 creates the Teacher Professional Development System, which establishes support and assessment mechanisms for teachers in training and novice teachers that must be implemented early, since the law also regulates the entry requirements for teacher training courses. For more information, see: https://registroycertificacionate.mineduc.cl/wp-content/uploads/sites/94/2018/03/Ley-N%C2%BA-20.-903-Sistema-de-Desarrollo-Profesional-Docente.pdf (accessed on 3 March 2022).

To successfully achieve this, evaluation instruments must be established to allow detection of students’ levelling needs and provide valuable information when defining support plans that can help to improve their quality of life [1,2].

It is important to highlight that the academic workload on student music teachers in the context of this study is quite high (nine courses per semester). In addition, although many students have access to free studies (by public policy), students do not receive an economic grant to help with their expenses. Moreover, music students’ musical backgrounds are quite heterogeneous [3,4]. All these factors can contribute to putting the students’ well-being at risk.

This research investigates whether psychological features related to the cognitive background of music students (musical sophistication) might be associated with well-being in adult music learning. From this perspective, by implementing a brief ten-question test—the Ollen Music Sophistication self-reporting questionnaire [5]—we aim to anticipate mental health issues that students may experience in their first year of music studies at the university. Thus this study highlights the importance of identifying students with a potential well-being risk in good time, and supporting them during their first year at university. We aim to provide evidence that students’ well-being can be enhanced through projects that resignify the use of music in their daily life from an academic perspective.

### 1.1. Musical Sophistication

Current knowledge of music abilities has traditionally been associated mainly with music practice and expertise [6,7]. Less attention has been paid to integrative approaches that consider the diversity of experiences that may be related with exposure to music. Therefore, in addition to the development of various measures of musicality [8,9], new ways of understanding this complex phenomenon have been established relatively recently. Musical sophistication constructs links of combined perceptual, theoretical or conceptual skills that can respond to this diversity in exposure to music [10]. Thus, Müllensiefen et al. [7] define musical sophistication as a “psychometric construct that can refer to musical skills, expertise, achievements, and related behaviors across a range of facets” (p. 2). It can be understood also, as pointed out by Hallam and Prince [11], as a synonym of musical ability or even general musicianship.

Ollen [5] introduced the musical sophistication index (OMSI) after carrying out an analysis of the literature on music abilities; from this she distinguished determining indicators that she then validated by using experts’ ratings. The OMSI is an integrated measure that must be understood as a whole; it would not make sense, according to the author, to analyze each question separately. It is a short questionnaire consisting of ten questions that can be answered by both musicians and non-musicians with various degrees of musical experience. The test has been used in different contexts and domains, for example, as a measure of musical expertise [12,13,14]. It has given rise to a significant number of related publications with considerable prevalence in music psychology literature. In Chile, which is the study context of the present research, it has been shown that the OMSI can predict cognitive performance in musicians and non-musicians, such as verbal working memory, fluid intelligence, processing speed, dividing attention, and cognitive inhibition [15].

Recently, Zhang and Schubert [16] compared the OMSI with the Goldsmith Musical Sophistication Index, a validated musical sophistication test that has been frequently cited in literature, showing that the OMSI single-rank item “Which title best describes you?” was a strong indicator for predicting the results of both tests, constituting a meaningful datum for assessment of musical sophistication. Gonzalez-Sanchez et al. [12] show, albeit with a very small sample, that musical skill (measured by the OMSI) can be predicted in cello and drum players by features of music performance, particularly smoothness of movement and the integration of sequential movements into single units (coarticulation), a skill related to music anticipation. In the Chilean context, the OMSI questionnaire was translated into Spanish [15] with a resulting estimate of Cronbach’s alpha = 0.77. The original data [5] showed similar reliability (=0.78).

Measurements of musical sophistication have thus provided a different perspective to the traditional view of musical abilities related to practice and experience, and at the same time have revealed new approaches to a more integrated understanding of what constitutes musical ability. We therefore believe that the heterogeneity presented by the cognitive backgrounds of music students should be evaluated considering musical experiences of various kinds. Indeed, these experiences could have an impact on students’ development during their first year at university, a critical period that has been analyzed through the results of much research in the field [17].

### 1.2. Music Students and Well-Being

Daily use of music can contribute to several positive effects that are currently being investigated by various disciplines [18]. These disciplinary fields could be strengthened if we knew in greater depth the impact that the daily use of music could have on people who decide to pursue a musical career. Despite the positive effects of music, musicians can also experience adverse effects linked to their profession, the origin of which is unknown in certain cases (e.g., tinnitus, focal dystonia). In fact, a high percentage of music students have health problems due to their profession (between 50 and 60% according to Demirbatir [19]). Indeed, musicians experience a high degree of anxiety in their career [20,21] and many of these deteriorating effects of music practice are attributable to their lifestyles when they were students [22].

It seems that most studies have a tendency to assume that “well-being benefits follow from music participation” [23] (p. 75). Zander et al. [24], in a longitudinal study, show that music students do in fact have greater psychological and well-being problems during their first two years at university compared to students on other degree courses. Similar results were obtained by Demirbatir [19], who observed higher levels of stress among first and second year university music students in Turkey. According to Ginsborg et al. [22], it is important that the problems that affect musicians should be monitored from the start, or from early practice stages onwards, not only to prevent harm and possible disorders, but also to positively influence their health and well-being.

The concept of well-being emerged from positive psychology. Martin Seligman [25] introduced a well-being model consisting of five components: Positive emotions, Engagement, Relationships, Meaning, and Accomplishment.

From a musical perspective, positive emotions could be related to a pleasant feeling after playing music, performing well in a lesson, or giving a successful performance in a concert. According to Seligman [25], all these types of sensations that make one ‘feel good’ are related to positive emotions. It is known that the subjective feeling of pleasure with music is regulated by the human dopaminergic system, which also has a role in memory and attention during learning [26]. On the other hand, negative emotions can influence the approach to music and learning. As mentioned previously, situations of stress and anxiety are very common in musicians, as well as stage fright or titrated fear of exposure. Negative emotions could also cause a deterioration in life satisfaction as well as in physical and psychological health [27,28,29]. Both positive and negative emotions could be also mediated by self-efficacy [30] and music engagement [29].

In music, engagement is essential: first with oneself, and then with others with whom one is playing, or with musical communities. Engagement exists equally in achieving a musical performance that respects the composer’s intent, or it can relate to conveying expressions of feeling committed to communicating something. The concept of flow has been investigated in music students by Smolej and Avsec [31], showing that those positive feelings, engaging complete involvement in an activity [32], are more related to emotional experiences than to cognitive well-being. From this perspective, the importance of developing enjoyable learning environments for students has been also highlighted [33,34].

Music practice and participation are intimately related with the PERMA framework [35]. Previous research has analyzed the different components of the PERMA model in professional musicians, establishing important observations from different approaches (qualitative and quantitative) [36,37]. The authors distinguish that the sense of identity acts in musicians as an “over-arching sustainer of well-being”; it is of transcendental importance for well-being both in professional musicians and in the process of educational transition. In turn, the sense of relationships is crucial for musicians since it is the key element in positive functioning, which implies a balance between the musician’s professional and personal experiences [37]. On the other hand, musicians also obtain higher scores in the dimensions of Positive Emotions, Relationships, and Meaning than the general population: Meaning in particular is the component in which musicians obtain the highest scores, with a parallel decrease in negative emotions. The Meaning component is indeed transcendental in music and artistic careers, since it is related to the sense of belonging to a culture, the sense of what it means to dedicate oneself to playing music or teaching, as well as knowing how to interpret the meaning of the work of a composer, i.e., what the composer wanted to communicate. Indeed, for Seligman [25], Meaning implies going beyond the self.

### 1.3. Rationale of the Study

The aim of this study was to explore the relationship between musical sophistication (measured through the OMSI) and students’ well-being.

From the perspective of the construct of well-being, music students who lack musical background may face greater difficulties in their educational transition (from high school to university) than their peers. To carry out the research, we set out to monitor first-year student music teachers for six months. The study integrates different approaches (qualitative and quantitative) and does not focus on academic performance alone. We were interested in understanding educational transition as a process where the goal of education is life fulfilment and not just professional success, as Seligman [25] states. We hoped to find clues to understanding the resignification processes of the daily use of music in an academic context. Indeed, as we mentioned previously, music students suffer higher levels of anxiety, fatigue, depression, or other psychological disorders compared to the general population [16,21,38]. There is clearly a need to better understand under what circumstances and in what contexts these problems occur. We therefore think it is important to investigate whether first year music students’ well-being could be associated with musical sophistication. Even though different constructs are involved, a possible link should be explored and discussed.

We also wanted to incorporate suggestions raised in previous research, particularly related to the importance of studying well-being from a qualitative approach, as well as the importance of studying sub-groups within the population whose well-being may be at greater risk [39].

## 2. Materials and Methods

### 2.1. Participants and Context

Twenty-five first year student music teachers from the same 2021 cohort participated in the study (16 male and 9 female). The average age was 20 years (SD = 3.72); 65.2% of the students came from government-subsidized private schools, 17.4% from public schools and 17.4% from private schools.

The music teaching degree at the PUCV Music Institute lasts 9 semesters, with an academic load of 9 courses per semester. Students do not have to take a special entry test (the entrance quotas are determined by their scores in the PDT University Transition Test and high school grades). The curriculum includes disciplinary, fundamental, and teacher training courses. In the first year, a large part of the courses are disciplinary. There are three professional practice stages throughout the study plan. Students of the institute stand out for their good marks at the national level in the test taken by student music teachers one year before graduating. Degree courses in music, composition, and musical performance (with its basic cycle) are also taught in the Music Institute.

### 2.2. Design

We performed a mixed exploratory sequential study [40] with one repeated measure (music sophistication). The design included measures of music sophistication, students’ well-being and academic performance (high school and first semester of university), complemented by qualitative semi-structured interviews. The data were collected over a six-month period.

### 2.3. Hypotheses

The theory posits that PERMA components (independent of one another) can contribute to personal development and a state of personal well-being [25]. Moreover, basic psychological needs must be satisfied in order to achieve good psychological development and personal well-being [41]. When those elements are not fulfilled, this may be reflected in problems of motivation and autonomy [42,43], as well as discomfort and frustration [44], which ultimately affect academic performance [45]. Previous research using self-reporting has shown that musical sophistication predicts visual, aural, and creative skills [8], as well as cognitive performance [15]. Thus, considering the theoretical background presented, in the present study we expected to find:

(1) That first year music students with poor musical backgrounds (measured through the OMSI) face greater difficulties in the educational transition process than their peers, from the perspective of the construct of well-being.
(2) An association between academic achievement and musical sophistication. On the one hand, this association could be based on previous musical knowledge and experiences, which should have an impact on students’ ability to develop more effective study strategies, thus mobilizing their previous knowledge. On the other hand, it could be based on students’ cognitive competencies (see Introduction).
(3) A relationship between the university transition test (in this study, PDT) and academic performance. In contrast, high school performance (in this study, NEM) should have less influence, since it is directly related to the student’s context and school culture, and is not a test of national scope. Indeed, previous research shows an association between PDT and academic performance in university students, which could be related to a socioeconomic factor [46].

From a qualitative point of view, the subjective representations of the students should help us to understand in greater depth whether the relationship between musical sophistication and well-being is connected with crucial processes such as the development of students’ identity or studying strategies.

### 2.4. Instruments

#### 2.4.1. Musical Sophistication

We applied the OMSI, a new measure developed as a result of Ollen’s thesis work. The music sophistication index includes ten factors (age of the individuals; individuals’ age at the initiation of musical activity; number of years that they have taken private instrument classes; number of years they practiced playing their primary instrument; amount of time currently invested in playing an instrument or singing; if they have studied music at the university level, or completed courses or degrees in music; their experience as musical composers; their attendance of concerts in the previous year; and their self-perception regarding professionalism in the discipline of music) and one global indicator of musical sophistication. The OMSI score (between 0 and 1000) indicates the probability that a respondent would be classified as “more musically sophisticated” by a music expert [12].

#### 2.4.2. Students’ Well-Being

We applied the PERMA-profiler [1] instrument, which contains 23 items categorized in five dimensions, each with three sub-items: (1) Positive emotions; (2) Engagement; (3) Positive Relationships; (4) Meaning; and (5) Accomplishment. The instrument also includes eight additional items: Negative Emotions (three items: sadness, anger, and anxiety); Self-perceived Physical Health (three items); one item assessing Overall Well-being and one for Loneliness. For the authors, the inclusion of negative emotions is important for considering both positive and negative elements of the mental health spectrum. The test was applied using a Likert-type scale from 1 to 10. An average score was calculated for each dimension (three sub-items each). The Spanish version of the instrument was validated in Chilean university students by Cobo-Rendón et al. [47], reporting consistency indices of the total scale and most of the components of α = 0.65 to 0.91 and ω = 0.66 to 0.92.

Sociodemographic information, university transition test (PDT), high school grades (NEM) and academic performance in the first semester at university were taken from an internal University database. The PDT instrument corresponds to the mean of mathematical and verbal comprehension abilities. The NEM corresponds to the mean score in all courses at high school over the previous 4 years.

### 2.5. Interviewing

Seven semi-structured individual interviews were conducted. Students were initially selected for interviews based on their musical sophistication score. We began with two students, and at the end of the semester five, new interviews were implemented. In the second round of interviews, the selection criterion was based on self-identity (Question number 10 in the OMSI). Interviews were implemented in order to approach students’ musical backgrounds based on the following perspectives: (1) to explore in greater depth what was stated in the musical sophistication questionnaire (e.g., self-reported musician rank); (2) students’ learning strategies, academic difficulties, and academic load; (3) previous experience in music in general or at school; and (4) emotional engagement, motivation, and well-being during the first six months of the course.

### 2.6. Data Analysis

Variables: (1) music sophistication index (one global indicator of musical sophistication); (2) PERMA-profiler well-being components including eight additional items (see Section 2.4.2); (3) academic performance, which included specific musical courses (piano, solfege, music history, choral practice); (4) university transition test (PDT); (5) high school grades (NEM).

A principal component analysis (PCA) of the quantitative data was carried out to determine the relationship between the variables studied, searching for particular strengths between musical sophistication, well-being, and academic achievement. The PCA produced some principal components (PCs), which are organized based on the amount of variance in the data explained by each component [48].

Qualitative data were assessed by means of comparative constant analysis, from which representative categories emerging from the discourse were individuated. In the first step (during the fourth month of university study), we wanted to explore whether previous experiences with music had an influence on the ongoing transition process between high school and higher education. This first exploratory step was carried out by comparing differences between more sophisticated students and less sophisticated students, focusing on previous musical experiences, academic difficulties, and learning strategies. This allowed us to deepen the relation, showing how these experiences determine certain practices and ways of approaching study. In the second step, and following our preliminary quantitative analysis, we explored whether students’ well-being is influenced by the university context and activities, considering emotional engagement, motivation and self-identity. This analysis allowed us to link observations from the interviews with the quantitative results, from which new categories emerged. The final code system allowed certain interpretative dimensions to be examined in greater depth to account for complementary, subjective aspects of the analysis, such as negative emotional impact (negative emotions), the development of identity as musicians, and the musical experiences of the musicians interviewed. The analysis was carried out with the qualitative analysis software NVivo 10 (QSR International, Burlington, USA)). Further triangulation of results (from qualitative and quantitative sources) was performed in order to better understand the different dimensions of well-being and their links to the educational needs of students with different musical backgrounds.

### 2.7. Procedure

The process was performed through: (1) initial testing of musical sophistication at the beginning of the academic period (in order to distinguish sample groups based on the test results); (2) gathering qualitative information on students’ educational needs during the academic period through interviews and the definition of representative categories of their well-being; (3) analyzing the academic results at the end of the first semester (results of discipline-related music courses); (4) re-application of the Ollen [5] musical sophistication questionnaire at the end of the first semester, to detect a possible continuing gap between students; (5) application of the PERMA-profiler as a measure of student well-being.

In the present study, measures of musical sophistication and well-being were administered in online versions, applied using the Google Forms platform.

## 3. Results

### 3.1. Descriptive Statistics

Descriptive statistics are presented in Table 1. The reliability of PERMA-profiler was calculated: Cronbach’s alpha = 0.87. We observed that the OMSI results were under 500 points on average, out of a maximum of 1000. According to Ollen’s (2006) criteria, this means that we can consider this group of students in general as musically unsophisticated. It must be noted that three students obtained more than 500 points in the OMSI, the highest score being 950. In the PERMA-profiler, the highest scores were engagement and negative emotions, which contrasts the results obtained by previous research in professional musicians [36]. On the other hand, the lowest scores correspond to physical health and accomplishment. In the PDT scores, only one student obtained a score below the national average, showing that these are outstanding students from an academic point of view, at least in language and mathematics (see Table 1).

### 3.2. Principal Components Analysis

**Figure 1:**
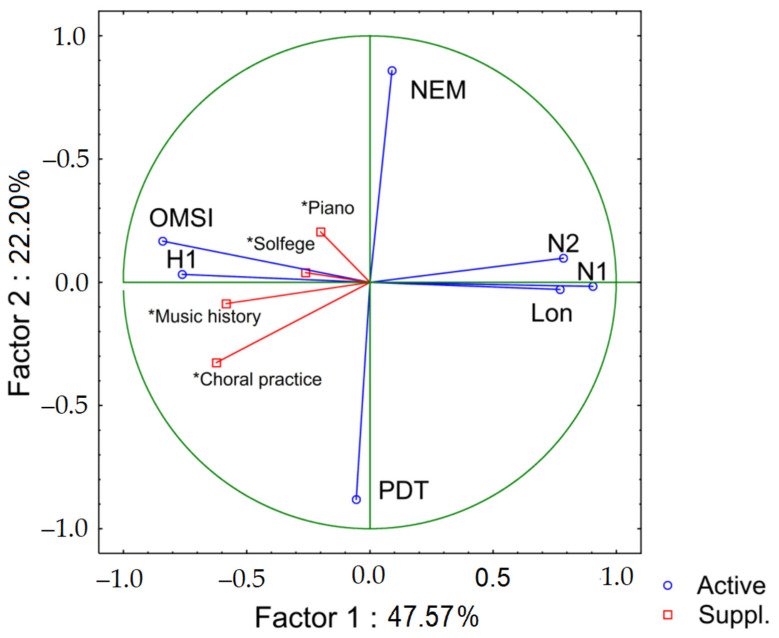
Principal components analysis.

In order to explore the relationship between the variables studied, we performed a PCA of those students who responded to the PERMA questionnaire with complete data (*n* = 18). We were interested in highlighting the variables that play a part in the transition process of student teachers, and that reflect the relationship between musical sophistication and well-being. According to their representation on the factorial plane, the following variables were selected: musical sophistication (OMSI), high school grades (NEM), University Transition Test (PDT) and the PERMA-profiler additional items: self-perceived physical health (H1), Loneliness (Lon), negative emotions of anxiety (N1) and anger (N2) (see Figure 1). As pointed out by Jolliffe [49], when the study is descriptive and the purpose is not inferential, it is possible to include variables with a certain degree of dependence in the PCA. The data were included without any culling or transformation. PCA was based on a correlation matrix.

Two principal axes can be distinguished from the PCA, one opposing musical sophistication and physical health (OMSI, H1), to negative emotions and loneliness (N1, N2, Lon), and the second opposing high school grades (NEM) to University Transition Test (PDT). The two axes explain 70% of the variance. The second axis is independent of most of the variables studied.

Academic performance variables were included as supplementary variables because of their weak representation in the factorial plane. The PCA shows that the academic performance variables are more closely related with the axis of musical sophistication, and in particular with positive self-perception of physical health.

The negative correlation between academic performance (solfege and history of music) and loneliness stands out. In turn, students who perform well in choral practice feel less anxious, less lonely, and have fewer feelings of anger (see Table 2). These results may be linked with teachers’ teaching methods or the evaluation mechanism, nevertheless, there is a common trend in that all the performance variables are grouped along the same axis. Otherwise, they correlate with each other in many cases. The strongest (negative) correlations are observed between OMSI and N1 (anxiety). The strongest (positive) correlations are observed between negative emotions (N1, N2) and loneliness (Lon). The OMSI and self-reported physical health (H1) are also significantly correlated.

It is important to highlight these results since the experience of singing (choral practice), as a direct musical expression, could improve well-being as pointed out in previous research [50]. However, this benefit would be selective since an association between the OMSI and negative emotions is observed, which indicates that there is a group of students that is excluded. The absence of association between these results and university entrance exams, or academic performance in high school, suggests that it is a tension that is generated as part of the transition process and mostly linked to previous knowledge in the musical field.

This tension is manifested at different levels. For example, it stands out that self-perception of physical health correlates negatively with anxiety. As pointed out in the introduction, a large percentage of musicians suffer from anxiety, which in case of performance (individual, group, or solo practice) is strongly related with the fear of negative evaluation [51]. It is also observed that anxiety is negatively associated with the OMSI. The foregoing shows that anxiety affects students at different levels, with a greater impact in those who have less previous musical experience. It could be that they feel more exposed than their peers, a condition related to feelings of loneliness and insecurity. This somatic and cognitive correlation has been previously described in both professional musicians [52] and adolescents [53], and is largely determined by social anxiety [51]. The foregoing is reaffirmed by the correlations observed between loneliness and anger, which suggests that this frustration is embodied, making sense of what we have already indicated regarding the feeling of physical health.

### 3.3. Self-Identity and Group Characterisation

As mentioned in the introduction, question number 10 of OMSI, “Which title best describes you?”, is an important item for assessing musicians’ self-identity and for comparing results with the Goldsmith Musical Sophistication Index [16]. In the first application of the questionnaire, 22.7% of the students declared themselves as a music-loving non-musician; 45.5% amateur musician; 27.2% serious amateur musician, and only one student self-assessed as a semi-professional musician. Between the first and the second application (at the beginning of the first year and six months later, respectively), we compared the answers to question number 10 of the test and found that three students had advanced from amateur musicians to serious amateur musicians, while the other students remained on the same level.

**Figure 2:**
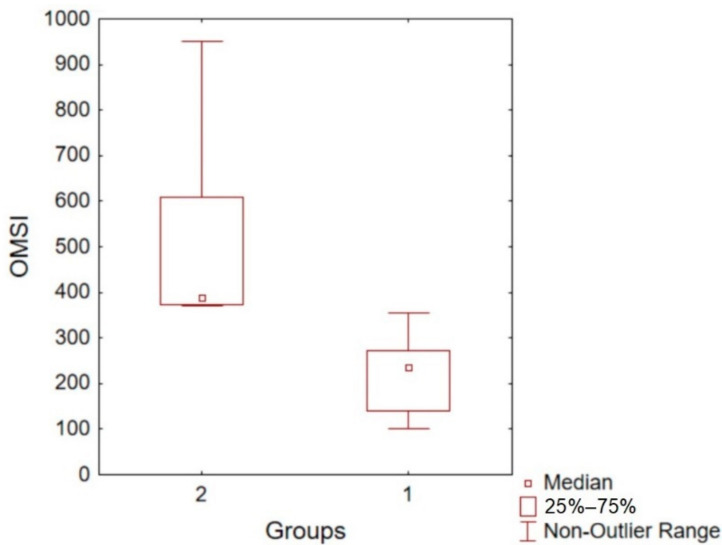
Boxplot for OMSI. Group 1 = less musically sophisticated; Group 2 = more musically sophisticated.

In order to explore the OMSI data, we proceeded to separate the sample into two groups based on their musical sophistication scores. The less sophisticated group is observed to be more homogeneous than the more sophisticated group (see Figure 2). Thus if the link between musical sophistication and well-being is strong, the less musically sophisticated group is very likely to be affected by similar psychological factors related with well-being. Given the small sample size, we investigated these implications by means of qualitative analysis (see next section).

### 3.4. Qualitative Analysis

In the first analysis, we compared the categories that emerge from the discourse of two students presenting opposite scores in the OMSI (see Table 3). The subsequent sections provide qualitative analyses by theme, including direct quotations from all the students in both rounds of interviews.

The purpose of the analysis was to identify, in the students’ own discourse, reflections about the dimensions of PERMA-profiler, musical sophistication, and study strategies. In parallel, we attempted to verify the extent to which the data reflected or contradicted the quantitative findings. The interviews allowed us to delve into certain aspects, such as negative and positive emotions, as well as the relationship between the trajectories of the students and their musical identity. In addition, other topics were developed, such as performance in secondary school and the students’ transition process. We draw attention to those situations that enabled students to resolve these tensions and improve their well-being.

Table 3 shows that the profiles of the two students differ in their depth of previous musical experiences. This seems to influence the way students perceive their relationship with music and their study strategies. The greater their wealth of experience, the more autonomous students feel, improving their capacity for self-regulation and responsibility in the study. In contrast, experiences that are less profound or less creative tend to be mainly reproductive, and are associated with a greater need for recognition or external validation. Moreover, in the latter case there is no real knowledge or appropriation of a musical instrument. In terms of self-esteem, the student with the higher score has a positive self-perception of himself, his work, and his academic performance; whereas the student with the lower score does not easily recognize the strengths of her musical work.

#### 3.4.1. Impact of Negative Emotions

The first dimension of analysis aims to account for the emotional aspect associated with studying to be a music teacher. When students are asked about their well-being in the course, emotions such as stress, pressure, and being overwhelmed by the academic load appear.

“I have had moments of depression, mainly due to the academic pressure; I don’t know. I am really having a bit of a hard time keeping up with the pace online, because the assignments and the questions and the deadlines keep piling up”

(E7)

Although anger is not directly mentioned in the reports, the students refer to associated concepts, with the feeling of frustration being one of the most recognized by the students when asked about the academic load.

“The very fact that it is not balanced (the academic load) ends up making it strenuous, very tiring at the end of the semester... like things speed up a lot, you realize that the semester is ending and that there are several things to teach and to evaluate (by teachers). That makes it stressful”

(E5)

As the student points out, academic pressure affects students’ emotional well-being. Difficulties in organizing their time and establishing study habits and strategies become an obstacle when it comes to developing positive emotions related to academic effort. It is striking that few students indicate having a well-defined, general study strategy throughout the semester.

“I think that now that I look back on it, like not experiencing it, there were weeks with a lot of stress. I mean, I don’t know if it was stress but a heavy [academic] workload, where the only thing that really mattered was handing in [the work]. It didn’t matter whether I slept or not, I had to hand in the work because it represented a grade. But there were also weeks that were totally relaxed and you could stay in bed or go for a walk, go out for a drink. But some weeks were like that and others…”

(E4)

The lack of time for non-academic activities also translates into being overwhelmed and frustrated, undermining the well-being of these students. These emotions peak at certain times of the semester, which is when the evaluations are squeezed together, so that the students also distinguish between periods of calm and academic overload.

“By the middle of the semester it’s more like—well, depending on the class—but more like, calm. And [you can concentrate on] how to find stability within the classes, how the evaluations work and everything related with that. And by this [later] stage of the semester, it is already pure stress, really a lot”

(E6)

It must be noted here that this research was carried out during the COVID-19 pandemic. The arrival of the pandemic, and the restrictive measures that obliged universities to teach courses online, were additional factors that affected these students. The idea of being stuck in front of a computer all day also contributed to the development of negative emotions. One of the interviewees indicated that studying alone had a negative impact on well-being during the confinement period. This modality not only affected students emotionally, but also made it difficult for them to study certain courses.

“Online, let’s say, it’s a little more complicated to deal with. Because in my case, for example, I don’t have classmates who are from here, or even from my region. So I find that I have to study completely alone. And studying solfege online, for example, I don’t know… I’ve tried it and the truth is that it’s not the same, it’s not the same as getting together with a couple of friends face-to-face and all practicing together”

(E7)

#### 3.4.2. Identity Development

In cultural studies, it is recognized that the development of identity is not linear. It is understood as “a rhizome of identity, not with a single root, but with multiple roots”, which is under constant construction and development [54] (p. 25). Identity or subjectivity is understood as a process through which the subject gives meaning to his/her experiences and environment through practices. Likewise, from this perspective, it is also understood that identities are positional; in other words, an identity can occupy different positions depending on the social space, and also subject to positions of power [55]. For example, in musicians there is a multi-identity development process related to the different roles and professional activities that a musician can perform in society, such as being a composer, a conductor, or a teacher [37]. Identity will be strongly linked to the sense of belonging to a group (in this case of artists and/or musicians) and to the course of the subjects’ lives. So the career of these students will shape their artistic identity.

During the interviews, the participants were asked about their different experiences and their self-definition as musicians. In their responses, some important elements were reflected in how they considered themselves as musicians, which in turn molded their identity.

“I feel like I have a good time playing… I’ve played in orchestras, I’ve accompanied singers, and I’ve played folk music, duos, and in some reggae bands. I don’t remember the rest. Once I went travelling and playing with a friend...”

(E3, amateur musician)

Experiences such as those reported by the student in the previous quotation are some of those mentioned by interviewees as important milestones in their career. However, when it comes to defining themselves as musicians, students outline certain criteria that would allow them to achieve that status.

The first element, as indicated above, is artistic experience and knowing a large repertoire of works.

“I would say I lack theory: knowing scales better, learning more, more works, but above all, experience”

(E7, serious amateur musician)

Thus, theoretical knowledge is also important, since it is recognized as an important element for self-definition as a musician in general by the students interviewed. Training is essential for this point.

“**Q:** What do you think is required to be a professional musician for example?

**A:** I think knowing how to do what I’m doing when I am performing music... improvisation or something like that” (E4, amateur musician)

“I think that is what you need—I don’t know if it is complete comprehension of knowledge—but rather the ability to apply everything I know, for example at the level of improvisation or composition, something like that”

(E6, serious amateur musician)

However, theoretical knowledge is not the only criterion when defining oneself as a musician; performance experience and improvisation and/or composition skills are also important, as indicated by the previous quotes, since this is how students demonstrate the implementation of their knowledge and what they have learnt from experience.

#### 3.4.3. Identity Development and Negative Emotions

As mentioned in the previous section, the development of artistic identity is related to the students’ experience. Thus, the greater the experience (public performances, composition, improvisation, performance on different instruments, and transmission of knowledge), the more their self-defined identity approaches that of a professional musician, generating a sense of security and confidence.

The following example tells the story of one of the students who points out that his experience as a member of musical groups—outside formal studies—has been helpful during the degree course, as he has greater mastery of concepts and fewer learning difficulties.

“I feel that, with all the experience that I have, I have an... I definitely have an advantage over my classmates. Er, I even have a sort of feeling that I am more or less at the same level as those who studied music, because in some fields, even theoretical ones, er... there are certain concepts that I have mastered. While those who haven’t studied music at all, or have no experience, find it much more difficult. So, yes, I do have an advantage because of my practical experience”

(E2, serious amateur musician)

It should be noted that, based on his practical experience, this student defines himself as a serious amateur musician, a category close to that of professional musician.

“Yes, a lot really. For example, in the case of reading [music], it’s not like I don’t get nervous, because of the presence of the teacher, but of course if I hadn’t given several performances during high school I’d probably get much more nervous and suffer much more”

(E6, serious amateur musician)

The same happens with this student—who also defines himself as a serious amateur musician—who has given several public performances. This type of experience allows students to face being tested by their teachers with greater confidence.

The opposite case is that of a student whose experience does not go beyond the music that she learned at school, where she was an outstanding student. However, her experience is limited to formal music studies.

“I feel that I am learning a lot, that it is not difficult for me to learn; but I feel that suddenly everything is very, very fast. I do not feel that the teachers go too fast, but because I have classmates who know much more and who come from the conservatory and so on, I can’t grasp everything so quickly in classes and I have to watch the classes again to understand certain concepts and things like that.

**Q:** And this causes you a certain difficulty? Maybe stress in some sense?

**A:** Yes, because during the class I kind of feel, I don’t know, inadequate, or that I can’t learn so quickly, and it frustrates me”

(E5, music-loving non-musician)

This student points out her difficulties in learning in certain classes and compares herself to her classmates with more experience, realizing that it is difficult to keep up with them. This situation causes her frustration and a feeling of inadequacy, which can undermine the development of her artistic identity. As shown in the quotation, the student in question defines herself as a music-loving non-musician, one of the categories furthest from self-identification as a professional musician.

The examples above show that practical experience is important for training and for self-definition as a professional.

#### 3.4.4. Experiential Taxonomy

Continuing with the importance of experience in the trajectories of the students interviewed, a relationship can be established between these experiences and the self-reported ranking as a musician in OMSI. Different levels of experience of the students interviewed will be reviewed below.

We observe the statement of a student whose experience refers to what she learned about music at her school. The interviewee points out that what she learned during her school years was helpful in music reading, developing her personality, or learning to play instruments. However, it has not been helpful in learning the content that she is seeing now as part of her higher studies.

“The truth is, no; I feel that—more than music courses—personality or playing an instrument obviously helps. But right now, that is not the main thing, because what they are teaching now is solfege, chords, and other things. But I feel that it helped me to read the notes on the stave, things like that; but it’s like an extracurricular activity, I don’t know if it’s considered part of learning”

(E5, music-loving non-musician)

In addition, there is also a relationship between the experience of this student with her self-definition in the music course. She defines herself as a music-loving non-musician and she has less practical experience and formal study than other classmates.

A second student has school experience similar to the previous case; however, she also sought forms of self-taught learning, where she could complement what she learned at school. As in the previous case, although these experiences have served to gain basic knowledge, she has had difficulty keeping up with some courses.

“For example, I learned to play the guitar on the internet like many people, but I was never taught at school. What we did at school… in music class there were always 10 of us out of 90, like between three courses we got together a music class and we were lucky if there were 10 or 15 of us. Then the teacher gave a piece of music to those of us who were there and said, ‘Right, play it’”.

**Q:** Do you feel that this disconnection with the musical education you had at school has affected you?

**A:** It has been difficult, but I have learned and now I can find my way round the stave, but it still takes a bit of an effort; I missed that at school. At least I am learning where the notes are and perhaps the rhythm and values of the notes. It might have been a bit easier if I had known that beforehand”

(E4, amateur musician)

During the interview, the student points out that starting university studies with greater prior knowledge would have facilitated her learning process. Unlike the previous case, this student defines herself as an amateur musician, which can be attributed to her self-taught training and her attendance at other types of courses outside school lessons.

In the following example, although the student’s experience is limited to school time, he has participated in orchestras, played different instruments, worked in sound engineering, and taken part in various public performances. All this gives him more background than the two previous cases.

“During school, the only thing I did was participate in a workshop that had an orchestra-type format, but there was electric bass, drums, a combination of everything. We had to travel to La Serena, Coquimbo, because they invited us from another school. I really loved that experience of the tour—and the music (…) Honestly, it helped me a lot because, as I say, experience is what forms the character of the musician. For someone who is just starting, performing in front of an audience is a bit more difficult than for someone who has already performed several times”

(E7, serious amateur musician)

The student acknowledges that his experience has helped forge his character. This has been an advantage when it comes to performing to an audience, which—in his opinion—is something difficult to face. In addition, this student works as conductor of two orchestras, which strengthens his training. His experiences have led him to define himself as a serious amateur musician, highlighting the importance of experience for the university course.

We see next the example of a student who has more training than that provided by her school, since she had a private teacher who helped her prepare to enter university. Within the framework of this experience she learned different contents that have helped her during her professional training.

“I had private classes with the same teacher that I mentioned before (...). With two classmates we decided to ask him if he could give us private classes, and we started to see content that both we and the teacher would have liked to cover in classes, but we were not allowed to… and to prepare ourselves at a better level for me at the university. When the teacher was still there, we had a music workshop that was practically like a band because there were very few of us. So of course we saw a lot of content, and although we didn’t see a lot of scores, we did see a lot of concepts that appear in the scores, and that is useful for interpreting a score with an instrument or with the voice. [That] has helped me a lot in what I do now in the different branches of the course”

(E6, serious amateur musician)

In addition to this experience of more formal studies and participation in a workshop in the context of her secondary education, the student has performed in public in various circumstances, which also helped her to control the nerves and pressure typical of this type of event. Thus, formal studies (outside what she learnt at school) and live performances have been elements that favored her identity as a musician.

The last example is a student who has vast experience in participating in musical groups and playing various instruments. This type of experience has been helpful for him to consider himself a serious amateur musician and to have the self-confidence to perform to an audience, and has also enabled him to do well in the different courses.

“I have a lot of experience playing in a musical group, so my experience goes that way, more on the practical than the theoretical side”

(E2, serious amateur musician)

Finally, we should highlight the importance of the possibility of knowledge transmission as part of professional identity. The greater the student’s experience, the greater his possibility of transmitting what he knows to others, and this is an important criterion for a person to consider himself a professional musician, as the following student points out.

“**Q:** What do you think you need, or what is it that gives you the authority to describe yourself as a professional musician for example?

**A:** I think knowing how to sight-read and play several instruments; because there will always be someone who specializes more in one instrument than in another… And I think that mastering the theory, like harmonies and things like that, that I don’t [understand]... I do understand them, but in my head... I couldn’t explain them to another person right now. So I feel that a professional musician can do it and explain them clearly”

(E5, music-loving non-musician)

#### 3.4.5. Well-Being and Positive Emotions

The students not only mention negative aspects or emotions associated with studying, but also positive aspects closely linked to well-being. In fact, throughout their musical experiences, enjoyment has been essential to their continual progress in music. The passion for music, whether listening, singing, or playing an instrument, has been a fundamental element during their experiences.

“I feel like I have a good time playing, that’s what I feel most when I play. I’ve played in orchestras, I’ve accompanied singers, and I’ve played folk music, duos, and in some reggae bands. I don’t remember the rest. Once I went traveling and playing with a friend, stuff like that with music”

(E3)

For some of these students, being in the course that they have always wanted to study enables them to cope better with the difficulties associated with the academic load. From this perspective, one aspect of the enjoyment of music that stands out is the social relationships and links that are established around musical practice. So playing instruments with other people, playing in a band, or just sharing experiences with others around music is a positive aspect.

“A: Just the social relationship: you go out, play, meet someone, talk for a while, have a drink, agree to play, play; then you meet another person who is a friend of a friend. When you play music it’s like developing a network of contacts with the same people you get to know. A friend of a friend invites you to play and there you can meet more people. And it’s like, in that way you get to know people by playing with them, or by playing alone on the street. Or by yourself, but people get to know you, that’s how it happens”

(E3)

“Also because of a feeling of community: like, it’s not just me studying. I have lots of colleagues by my side who are experiencing the same as me; and if I’m experiencing it and feeling good, why shouldn’t the rest [do the same]?”

(E5)

## 4. Discussion

The present study analyzed the link between musical sophistication and the well-being of first year student music teachers. We observed, from qualitative data, that more musically sophisticated students are better able to organize their time, have a more positive attitude towards their academic achievements, and present more effective identification of self-strengths and positive perception of learning and music-related activities. These behaviors are related with self-determination and well-being [8,41]. In contrast, the less musically sophisticated group showed lower self-confidence and less efficient learning strategies, factors related to poor well-being in university students in Chile [56]. In the context of this study, less musically sophisticated students seem to be more exposed to having poorer personal well-being in the educational transition between high school and university.

The PCA results agree with the above and confirm our hypothesis, since an association between musical sophistication and student well-being is clearly observed at the factorial level. Although it is not possible to establish a causal relationship, having a weaker musical background prior to entering university is associated with more pronounced negative emotions and feelings of loneliness. On the other hand, the most musically sophisticated students declare that they have better physical health, and greater possibilities of better academic performance.

### 4.1. Educational Transition and Musicians’ Identity

We observed a strong link between the artistic experiences of students and the construction of their identity as musicians. When this link exists, we find a sense of security and confidence related to both academic and musical performances. Thus, a dialectic relationship can be established between artistic experience, identity, and self-confidence.

From the perspective of educational transition, analysis of the interviews also confirms that a poorer experience in high school is negatively perceived by student music teachers when faced with musical activities at university, which could have a psychological impact. Swart [57] argued that a mutual inter-relationship between self-esteem, identity, and the effectiveness of musical communication exists in aspiring musicians. Our results are consistent to some extent with these observations, as negative emotions are more present in students with a less sophisticated musical background. Indeed, these students also feel disadvantaged compared to their peers, and this has a negative influence on the development of their identity, as well as making them feel more insecure and less confident. We observed that theoretical knowledge is not the only criterion for defining oneself as a musician; performance experience and improvisation and/or composition skills are also important, as indicated by the quotations cited above, since it is in them that the implementation of knowledge and what has been learned from experience is demonstrated. Indeed, Burland [58], in a longitudinal study with undergraduate music students, shows that the factors related to the development of identity and coping strategies would have a greater impact on the transition processes between school and higher education in music studies.

Identity transitions are quite gradual; in fact we observed only three students who changed their self-perception in Question 10 of the OMSI (Which title best describes you?) from amateur musician to serious amateur musician after one university semester. These three students did in fact obtain higher than average scores in the OMSI, one of them being one of only two participants to who obtained a score of over 500 points in the OMSI; in other words, according to Ollen’s criteria, this student could be considered musically sophisticated. In this context, and considering the three-component model (skill, identity, and predisposition) for musicians defined by Zhang et al. [59], it could be concluded that musical predisposition (revealed by OMSI) would be decisive for the construction of the other factors, i.e., skills (placed on the predisposition axis and related with academic performance) and identity.

### 4.2. Why Negative Emotions?

The information collected in the interviews shows that students have difficulties in organizing their time and establishing study habits and strategies. This generates problems of anxiety, frustration, and feeling overwhelmed, negative emotions that in our study show a negative correlation with the results of the OMSI. Similar results have been observed in a previous study in science and technology university students; they show a relationship between the perception of academic load and negative emotions [60]. The novelty of our study is that it explores the relationship between these perceptions and the previous experiences of the students.

The feeling of loneliness is also negatively correlated with the OMSI score. In the interviews, we observed that this feeling was related with having to study online due to the pandemic. Students who are not from the Valparaíso Region (where the university is located) were told to establish relationships with their peers in order to study cooperatively. In addition, many students who have more previous musical experience belong to musical groups and have more opportunities to play together with others, which less sophisticated students probably do not yet achieve—and may not feel qualified to do.

This is a fundamental finding, and in the interviews, students highlighted the importance of developing relationships with others around music as a positive aspect for their well-being.

The feeling of anxiety (In general, how often do you feel anxious?) is a negative emotion that, together with engagement (How often does time go by very quickly when you are doing something you enjoy?) presents the highest average scores in the PERMA-profiler. Although the literature shows a relationship between music as a profession and anxiety (see Introduction), which can even form part of the dimensions of a musician’s personality [61], the information from the interviews cited above provides new clues to interpret the data. In this case, we refer to when the students mention that they do not feel capable, which, together with the impact of the academic load, hinders the process of developing their identity as a musician. Despite the above, it can be deduced that the students want to continue with their studies and their professional vocation, which is highlighted by their high levels of engagement. Conversely, previous research in active professional classical musicians has shown that Positive emotions, Relationships, and Meaning are on average the strongest determining factors for well-being [36].

In summary, negative emotions emerge when there is an imbalance between academic activities and non-academic activities, particularly those related to artistic experiences, or even when it is not possible to grow in the artistic environment. Indeed, academic pressure can be increased by the lack of effective study habits and strategies. This tension seems to appear also when students compare themselves with their peers, as mentioned in the previous section.

### 4.3. Academic Perspective

Qualitative data show that the fact of feeling good playing music and doing what they like generates positive emotions in students, which protects them from anxiety. On the contrary, those students who are not very satisfied with their performance and have a lower tolerance for frustration, or low self-esteem, would be more exposed to feeling anxiety. These findings are consistent with the quantitative results

The variables associated with academic performance are located on the axis of musical sophistication in the PCA. This may be consistent, to a degree at least, with the results of Porflitt and Rosas [15,62] in the Chilean context, who reported an association between OMSI result and executive functions. On the other hand, the independence of academic performance from scores in the university transition tests (PDT) and high school grades (NEM) is striking. These variables have been associated with academic performance in the Chilean context [63,64], and can be attributed in part to socioeconomic inequality [46]. Because 65.2% of the participants in our sample come from subsidized schools (a mid-point between public and private schools), it is difficult to argue about a bias of this type, and the limits of the present work do not allow us to reach further conclusions. Another possible explanation could be the fact that the academic scores of students who obtain admission to music courses are among the highest at the university, which could bring an important academic advantage. On the other hand, another important aspect of the axis of musical sophistication is the overlap between self-perception of physical health (H1 in the PCA) and academic performance. As a result—all other factors being equal—students feel that they have better physical health when they perform well; they feel less anxious and less alone. Perhaps behind this personal perception of good health and mastery of certain musical skills lie better personal growth, purposeful engagement and self-development, and factors related to eudaimonic well-being [65].

Turning to the factors related with the university transition tests (PDT), as we mentioned previously, there is evidence that results are influenced by socioeconomic factors. The NEM score was implemented in Chile precisely to avoid this bias in university selection; it equates to the average of the individual’s high school grades (a kind of meritocracy index). For this reason, the negative correlation between the two scores (PDT and NEM) in the PCA should not surprise us. However, it is rather interesting that both measures are independent of musical sophistication and negative emotions.

From this perspective, the music course could be considered as a cultural meeting place that apparently manages to transcend certain barriers. Indeed, we may note that the majority of the students in our sample came from subsidized schools, a more heterogeneous system lying between public and private education. We are constantly surprised by the number of students from these apparently neutral schools who present greater previous musical experience; this could be interpreted from the point of view of cultural advantage (e.g., through Bordieu’s class theory).

In any case, this difference in Ollen’s terms [5] is not extreme; in fact, we observed that only two students in our study exceeded 500 points in the OMSI test. From the foregoing, we could say that, despite being adults and music students, very few of them have had significant musical experience. The relevance of this study, therefore, lies in the fact that it explores musical learning in adulthood in university students who generally present low musical sophistication according to Ollen’s criteria. This type of situation is very particular and occurs in certain contexts or countries. The important point for the present purpose is that when we divided our sample into two groups based on musical sophistication scores, the less sophisticated group was more homogeneous than the more sophisticated group (see Figure 2). In turn, greater musical sophistication opens up a range of possibilities, since the type of musical experiences or musical abilities begins to expand, presenting much greater variability. It seems that one experience leads to another, and there is probably significant growth from certain basic experiences. For this reason, it is essential that school pupils should have the opportunity to develop in the musical field. Indeed, in the comparative table (Table 3) we observe that a large part of the differences between interviewees are explained by the depth with which previous musical experiences are accessed. Based on this, we may deduce that the less the depth of these musical experiences, the smaller the possibility of knowledge transfer [66]. This enables us to understand why more musically sophisticated students can develop efficient study strategies, and have a more creative approach to music. Indeed, the study by Fiedler and Müllensiefen [67] shows that higher developmental trajectories or interest in music over time “as a school subject” is correlated with musically active students, and especially students from schools that apply a selection process. What this indicates in effect, as we have suggested previously, is that the level of access to musical activities entails benefits that influence the formative paths of the students, which according to the authors would be related with self-concept and musical sophistication.

## 5. Limitations of the Present Research

Given that the emphasis of this research was on monitoring the same cohort of student music teachers, we believe that the size of the sample is a possible limitation of the study. Another point to note is that the research was carried out under pandemic conditions, therefore classes during 2021 were carried out mainly online, with some courses in hybrid format and very few in a traditional classroom setting. We believe that the results might be different in a non-virtual situation. 

## 6. Conclusions

In this research, we addressed the well-being of student music teachers during their transition process from high school to university according to their level of musical sophistication. A Mixed Methods approach led us to a better understanding of important psychological features of music learning in adults, and we observed complementary results between qualitative and quantitative perspectives.

From a quantitative perspective, the association between musical sophistication and well-being is highlighted by its independence from both university entrance tests (PDT) and high school grades (NEM). In effect, what is mainly affecting the students’ transition process is the problem of access to disciplinary knowledge and therefore the consolidation of their identity as musicians. At least during the period of monitoring in this study, we observed important psychological issues related to students’ well-being rather than situations of academic failure. Consequently, feelings of loneliness, anger, and particularly anxiety affect those students with less musical background to a greater extent, as they probably feel more exposed, and experience fear of negative evaluation [51].

From a qualitative perspective, students with low musical sophistication find that the pace of study is too fast and feel overwhelmed or frustrated when they cannot fulfil their obligations. This group is more homogeneous and their previous experiences with music are less profound. This in turn is related to lower autonomy and self-esteem, incipient self-regulation, insecurity, and difficulty in developing effective study strategies. In addition, they need more external validation, since their previous experiences have been more reproductive and less creative. In contrast, the more musically sophisticated students are more autonomous, more aware of their progress, and more easily able to identify their achievements. They develop a musical identity more fluidly and they feel more confident, which allows them to change their self-perception of musical identity more easily. Thus, from a pedagogical point of view (as future teachers), their professional identity can be founded more firmly, since feeling capable of teaching something is a factor that students consider relevant for being a professional musician.

In general, academic pressure affects both groups, generating stress in students, as has also been observed in other university contexts [68,69]. Playing and enjoying music, as well as being able to study a career they always dreamed of, are very important factors that generate positive emotions. Indeed, these experiences are significant since they allow students to interact with other musicians and audiences; from the point of view of self-determination theory, relatedness is important for achieving a state of well-being. Moreover, artistic experience and knowing a large repertoire of works emerges as one of the main axes of the development of self-perception as a musician, along with theoretical knowledge.

Turning to the effects of the pandemic, the main points that emerge refer to the difficulties involved in studying alone or working long hours in front of a computer, as well as not having the possibility of forming musical groups among classmates or developing in non-academic environments. These activities are important, as we have pointed out, because they are related to positive emotions and well-being.

In summary, we show that the learning processes of students with less previous musical experience are more complex and are associated with prevalence of negative emotions and difficulties in the development of their identity as musicians. Considering that the present study was carried out with future music teachers, it is important to consider that it is likely that the negative emotions that accompany musical learning may persist or be transmitted, depending on the nature of the teachers’ beliefs [70]. Indeed, for there to be a change in epistemological beliefs, very particular conditions are required, where psychological aspects are articulated within a particular context of social interaction [71]. Without these elements, which largely depend on the learning environment, future teachers may not fully experience well-being in their professional future, as has been reported in recent research related to context of studying singing [50]. It is important, then, from a didactic point of view, to consider in greater depth the nature of these students’ previous experiences, to strengthen support networks among peers, and to foment equal opportunities for teaching of the arts at school.

## Figures and Tables

**Table 1:** Descriptive statistics.

Variables	Mean	SD	95% CI	Shapiro–Wilk: W(p)	*n*
OMSI	346.73	198.23	[261–432.47]	0.91(0.071)	24
P	5.82	1.76	[5–6.64]	0.97(0.826)	20
E	6.87	1.57	[6.13–7.6]	94(0.275)	20
R	6.23	1.9	[5.34–7.12]	0.95(0.332)	20
M	5.95	2.29	[4.88–7.02]	0.89(0.032)	20
A	5.52	2.07	[4.55–6.49]	0.92(0.105)	20
N	6.62	1.77	[5.79–7.45]	0.93(0.15)	20
H	5.27	2.37	[4.16–6.38]	0.95(0.391)	20
L	5.95	2.34	[4.82–7.08]	0.95(0.404)	20
NEM	668.42	74.8	[637.54–699.29]	0.93(0.079)	25
PDT	606.7	62.13	[581.4–631.92]	0.98(0.79)	25

Note. CI = confidence interval; OMSI = Ollen musical sophistication index; P = positive emotion; E = engagement; R = relationships; M = meaning; A = accomplishment: N = negative emotions; H = physical health; L = loneliness; NEM = high school grades; PDT = university transition test.

**Table 2:** Correlation matrix.

Variables	M	SD	1	2	3	4	5	6	7	*8*	*9*	*10*	*11*
1. OMSI	365.38	222.95	1	0.56 *	−0.01	−0.12	−0.70 **	−0.59*	−0.57 *	0.41	−0.09	0.14	0.45
2. H1	5.88	2.39	0.56*	1	−0.05	0.02	−0.68 **	−0.44	−0.43	0.38	0.35	0.20	0.41
3. NEM	689.21	79.85	−0.01	−0.05	1	−0.53 *	0.14	0.09	−0.07	−0.18	0.41	0.17	−0.16
4. PDT	609.75	62.8	−0.12	0.02	−0.53 *	1	0.04	−0.14	−0.13	0.45	0.02	0.13	0.08
5. N1	8.5	1.26	−0.70 **	−0.68 **	0.14	0.04	1	0.64**	0.63 **	−0.50 *	−0.14	−0.11	−0.45
6. N2	5.25	2.57	−0.59 **	−0.44	0.09	−0.14	0.64 **	1	0.53 *	−0.65 **	−0.19	−0.15	−0.41
7. Lon	5.81	2.79	−0.57 *	−0.43	−0.07	−0.13	0.63 **	0.53*	1	−0.57 *	−0.30	−0.51 *	−0.65 **
*8. ChoralP*	6.33	0.67	0.41	0.38	−0.18	0.45	−0.50 *	−0.65**	−0.57 **	1	0.34	0.37	0.69 **
*9. Piano*	6.58	0.43	−0.09	0.35	0.41	0.02	−0.14	−0.19	−0.30	0.34	1	0.50 *	0.41
*10. Solfege*	6.29	0.55	0.14	0.20	0.17	0.13	−0.11	−0.15	−0.51 *	0.37	0.50*	1	0.55 *
*11. MusH*	5.64	0.71	0.45	0.41	−0.16	0.08	−0.45	−0.41	−0.65 **	0.69 **	0.41	0.55 *	1

Note. *ChoralP* = choral practice; *MusH* = music history; *8*–*11* = supplementary variables; * *p* < 0.05, ** *p* < 0.01.

**Table 3:** Emerging categories of verbalizations and comparative synthesis between interviewees with different degrees of musical sophistication.

Categories	Student 2. OMSI = 273	Student 1. OMSI = 14
Autonomy	Through listening and participation in groups	Through inquiry without a teaching figure
Self-regulation	Use of study strategies linked to values of responsibility (consolidated)	Study linked to motivation (not consolidated)
Music performance in groups	General experience in groups of diverse nature and repertoire	Experience in extracurricular groups, reduced repertoire, and concentrating on certain works
Instrument practice and transfer	Learning of various instruments with the possibility of playing them in different pieces and repertoires	Generally learning of instruments exclusively applicable to certain specific pieces
Instrument knowledge	Understanding of the sound and technical characteristics of the instrument	Rather operational or superficial approach
Self-Esteem	Positive self-perception of academic achievement and performance, identification of strengths, assessment of learning and musical work	Negative self-perception, little recognition of learning, and academic performance; Identifies strengths, but not always related to their musical work
Breadth of repertoire	Broad repertoire consumed, with diversity of genres and musical instances	Small repertoire consumed, little variety of genres
Musical approach in childhood	Through playing and exploration (creative approach)	Through imitation of exponents/models (reproductive approach)

Note. Student 1. OMSI = First interview with OMSI score of 14; Student 2. OMSI = Second interview with OMSI score of 273.

## Data Availability

The data presented in this study are available upon request from the corresponding author. Following the ethical obligations established by Law No. 20.120 on scientific research in humans, the data are not publicly available to ensure the privacy and anonymity of the participants.
